# Kinetic Analysis of Swimming Flip-Turn Push-Off Techniques

**DOI:** 10.3390/sports7020032

**Published:** 2019-01-28

**Authors:** Wendi Weimar, Andrea Sumner, Braden Romer, John Fox, Jared Rehm, Brandi Decoux, Jay Patel

**Affiliations:** School of Kinesiology, Auburn University, Auburn, AL 36849, USA; amsumner13@gmail.com (A.S.); bromer@highpoint.edu (B.R.); jfox@methodist.edu (J.F.); jrehm@hawks.huntingdon.edu (J.R.); bed0012@tigermail.auburn.edu (B.D.); jay.patel@palmettohealth.org (J.P.)

**Keywords:** freestyle, countermovement, velocity, impulse, peak force

## Abstract

The aims of the present study were to examine the influences of different push-off techniques on kinetic and kinematic parameters both in and out of the water. The two techniques were: (1) a push off that was characterized by rapid extension of knees and hips towards the wall, prior to contact (i.e., no countermovement), and, (2) where the swimmer glides into the wall, letting the wall flex the knees in an approximate countermovement or eccentric phase. Twenty trained male and female freestyle swimmers (age 26.1 ± 9.9 years, height 1.61 ± 0.04 m, and weight 65.6 ± 19.3 kg) participated. Data were analyzed by employing two (i.e., land and water) 3 (variables of interest) x 2 (push-off type) repeated measures ANOVAs with the alpha level set a priori at 05. Results indicated that there were significant main effects for peak perpendicular force (*p* < 0.001), perpendicular impulse (*p* = 0.018), and velocity at 2.5 m (*p* = 0.005) on land. However, no significant effects were found between techniques in the water trials. As many of the participants were master swimmers, it is possible that they were unable to approach the wall in the water at the requisite speed to elicit a benefit from the countermovement.

## 1. Introduction

Swimming is growing in popularity and the differences in podium positions are being determined by smaller and smaller margins. As a result, athletes, coaches, and researchers are looking for the slightest edge in each component of the swim race. A swimming race is comprised of the start, the strokes, and the turns. Previous literature has suggested that turns comprise of up to 30% of the race and that turn times even determined the bronze medal podium standings of the 2000 Sydney Olympics [1]. As important as turns seem to be to swimming races, it is surprising that much of the attention to swim races often focuses on the physiological costs and mechanics of stroke techniques, yet relatively little literature exists regarding the turn. In considering the flip turn, also called a short axis turn or tumble turn, the components include the approach, rotation, wall contact, glide, underwater propulsion, and stroke resumption phases [2]. Of these components, the wall contact phase is of utmost importance as it is the only time during which force can be developed. As a turn requires the swimmer to change direction by 180°, the wall contact portion is responsible for stopping the swimmer as well as generating the initial velocity immediately off the wall. Further, the velocity after the flip turn can be a major determining factor between the final positions in the medal stand [3].

There is two primary push off techniques that occur after the rotation during the front crawl stroke. One technique is characterized by a tight tuck and rapid extension of knees and hips toward the wall, prior to contact. This technique usually results in a forceful push-off, characterized by minimal wall contact time, and will be referred to as the no countermovement turn. The second push off technique more closely resembles a vertical jump. In this case, the swimmer rotates, touches the wall with their feet, and continues to proceed toward the wall. The feet of the swimmer stay in contact with the wall, and the knees bend as the swimmer approaches the wall. Once the knees flex, the swimmer then forcefully pushes off the wall. As this motion mimics a countermovement vertical jump, it will be referred to as the countermovement turn. While Blanksby et al. (1996a) has suggested that a quicker rebound may better exploit elastic energy [4], previous literature has indicated that countermovement jumps use stored elastic strain energy to achieve greater jump heights than jumps without countermovement [5].

The countermovement vertical jump has been divided into three components: unloading, eccentric and concentric phases [6]. The interesting thing about the countermovement flip turn is that it has only two of the three components. The countermovement turn lacks the unloading phase, which has been said to be the interval over which gravitational potential energy is being converted to kinetic energy, which is then used to demand the eccentric portion of the motion and develop elastic strain energy [6,7]. Previous literature has indicated that the faster a person reaches the lowest center of mass position, the higher the jump that is achieved [8]. This would suggest that in the countermovement flip turn, the faster that a swimmer could achieve a position of the center of mass closest to the wall, the greater the velocity off the wall that could be achieved. Therefore, the velocity with which a swimmer approaches the wall influences the development of elastic strain energy. In an attempt to eliminate this as a factor, a land version of the push off was conducted in the present study.

To ensure that the participants were producing a force profile that would provide for the inclusion of the stretch shortening cycle, a visual inspection of the force time curves was conducted. The force profiles produced in the present study follow the same general pattern as those produced in vertical jump research, with the exception of the unloading phase [8]. During the land condition, the eccentric and concentric phases are recognizable. However, in the water condition, it appears that more time is spent in the concentric phase than in dryland condition. It is reasonable to conclude that during the water conditions, the drag contributed to both the shorter eccentric and longer concentric phases. During the eccentric phase, the water would aid the swimmer in slowing the movement toward the wall, and during the concentric phase, the drag would provide increased resistance.

In a freestyle flip-turn, the eccentric phase consists of the initial wall impact and any countermovement when the swimmer is still moving towards the wall. The countermovement considered for this study was flexion of the knees and hips in addition to dorsiflexion at the ankle joints. The active force production phase consisted of the concentric contraction of the plantar flexors, quadriceps, and hip extensors in order to create velocity away from the wall. This phase is composed of a forceful extension about the knees and hips and plantar flexion at the ankles in the horizontal direction, which in turn, would provide the swimmer more force during the push-off [9] than during a no countermovement push off technique. The pre-loading in the muscle and associated tissue permits a faster contraction with a greater force than for a muscle that has not been pre-stretched [3].

Previous literature has indicated that advantageous turn times are predicted by peak force, with greater peak forces yielding the best times [4,10]. Lyttle et al. (1999) expanded on these findings and found that it was more advantageous to reach peak force in a more gradual manner so that peak force occurred close to the feet leaving the wall as this is the point at which the swimmer would be in the most streamlined position [11]. The most streamline position would be most advantageous because the drag would be minimized at the point where the velocity would be greatest. The gradual development of force naturally leads to the role of impulse. It may seem that the shortest wall contact time may be optimal; however, this may not allow for maximum force development [11]. In fact, Tourny-Chollet et al. (2002) has indicated that longer contact times demonstrated an increase in push-off speed [12]. Furthermore, the peak force data and impulse data from previous research has been quite inconsistent. Specifically, previous work has reported peak force from 535.9 N [3] to 1189.6 N [11], and impulse from 150.6 Ns [3] to 204 Ns [11].

The aim of this project was to compare the influence of different push-off techniques of the freestyle flip-turn on kinetic and kinematic parameters, investigating both in and out of the water. The land condition employed in this project allowed the researchers to control the orientation of the swimmer as they approached the wall and to assure that the swimmers achieved the appropriate body alignments per technique. This project endeavoured to gain a better understanding of the push off and provide athletes information to aid in the selection of a preferred technique during competition or recreational swimming. It was hypothesized that the countermovement technique would yield a greater perpendicular impulse (perpendicular to the wall) and greater velocity at 2.5 m after the push off on land and in the water.

## 2. Materials and Methods

All testing protocols were approved by the university’s institutional review board and each participant provided voluntary consent. Twenty trained, master, and recreational swimmers who were currently competing in freestyle served as participants (N = 20; 8 males, 12 females). Current participation in competitive swimming was ensured through a questionnaire. Following the securing of informed consent, demographic data were collected (age = 26.1 ± 9.9 years, height = 1.61 ± 04 m, and weight = 65.6 ± 19.3 kg) and a health screening questionnaire was administered to eliminate any participants that had sustained a performance limiting injury within the last six months.

### 2.1. Procedures

This study was conducted both on land and in the pool. The equipment used for both conditions were nearly identical with the exception of an underwater video camera (Helmet Camera, HC-1 NTSC Camera, Fredricksburg, VA, USA) used to analyze proper push off techniques while in the pool. Each participant completed 3 trials of each push-off technique on land and in the pool. The push-off techniques were termed no countermovement or countermovement. During the no countermovement technique, the swimmers were asked to extend their legs forcefully toward the wall prior to contact. During the countermovement technique, the swimmers were asked to make contact with the wall and allow the momentum of the approach to flex their knees, before forcefully pushing off of the wall.

**Figure 1:**
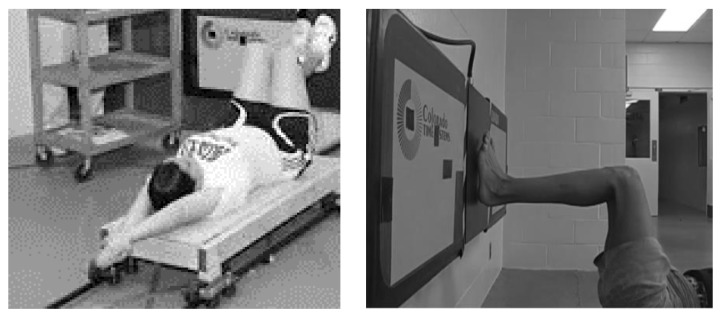
Instrumentation set-up for on land data collection.

For the land trials, the swimmers were supine on a sled which was placed on a railing system (Figure 1). The swimmers were pulled toward the wall with elastic tubing which was stretched to the same length for each swimmer. An AMTI Underwater Force Platform (WP6-7 1000, Advanced Medical Technology, Inc. Watertown, MA, USA) was affixed to the side of a wall and the two push off techniques were investigated. A camera was placed with a field of view that was perpendicular to the turn wall, in such a position as to ensure that 90° of knee and hip flexion angles were reached while the swimmer was in contact with the force platform, particularly during the countermovement trials. The swimmers completed 3 trials for each technique. The force platform was used to determine peak force and impulse. Force platform data were analyzed using AMTI NetForce software (Advanced Medical Technology, Inc., Watertown, MA, USA).

For the pool trials, the same instrumentation set-up was repeated. The identical waterproof force platform was affixed to the side of the pool and the two push off techniques were investigated. An underwater camera was affixed so that the field of view was perpendicular to the turn wall. This was achieved in such a position to ensure that 90° of knee and hip flexion angles were reached while the swimmer was in contact with the force platform, particularly during the countermovement trials. In addition, a Canon 3CCD digital video camcorder (GL2 NTSC, Canon, Lake Success, New York, NY, USA) was set at 2.5 m to allow the calculation of velocity. The swimmers completed three trials for each technique. They were instructed to begin at least 10 m away from the wall, to swim at their preferred race pace, approach the wall and perform the requested flip turn. They were further instructed to maintain their streamline position after pushing off the wall. There was no kicking or arm motion allowed. To determine the velocity of the swimmer at the 2.5 m mark, a known reference distance was hung above the middle of the lane at the 2.5 m distance from the wall. The position of the swimmer was noted three frames before and three frames after the 2.5 m marker. Utilizing the software program Dartfish^®^, the reference distance, position of the swimmer and the time were used to calculate the velocity.

### 2.2. Statistical Analyses

Data were analyzed using PASW 19 for Windows (SPSS, Chicago, IL, USA). The analysis included two (i.e., land and water), 3 (i.e., variables of interest) × 2 (i.e., push-off type) repeated measures ANOVAs with the alpha level set a priori at <.05. The variables of interest were force, impulse, and velocity at 2.5 m.

## 3. Results

**Figure 2:**
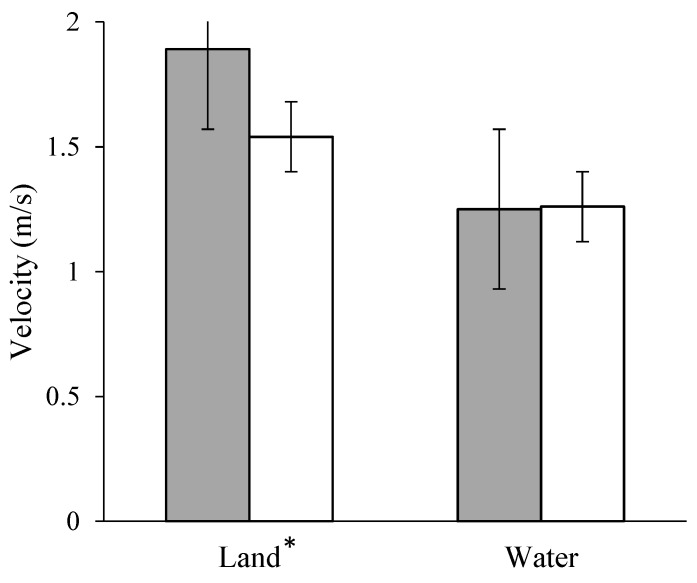
Velocity at 2.5 m from the wall on land and in water, the * indicates that there is significant differences within this condition.

**Figure 3:**
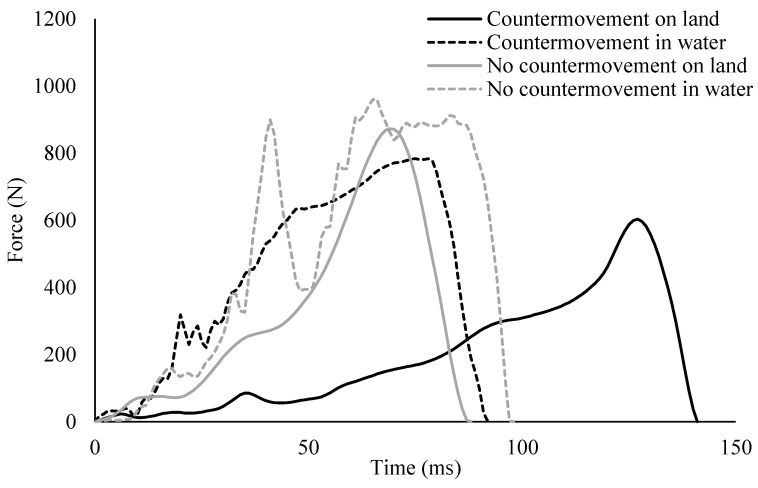
Representative force profiles during the wall-contact phase for the countermovement and no countermovement push-off techniques on land and in water.

The countermovement technique (1.90 ± 0.48 m/s) yielded a significantly larger velocity at 2.5 m than did the no countermovement technique (1.54 ± 0.31 m/s) (*F*(1,19) = 10.23; *p* = 0.005; 95% CI {0.13, 0.60}) (Figure 2). On land, the no countermovement technique (1075.5 ± 274.9 N) produced forces that were significantly larger than the countermovement technique (854.5 ± 310.5 N), (*F*(1,19) = 32.58; *p* < 0.001; 95% CI (−302.0, −140.0)) (Figure 3). However, the impulse for the countermovement technique (227.4 ± 56.5 Ns) was significantly larger than the no countermovement technique (198.8 ± 29.4 Ns), (*F*(1,19) = 6.76; *p* = 0.018; 95% CI {−52.23878, 5.52317}) (Table 1). There were no significant differences between the techniques for any of the variables in the water condition.

## 4. Discussion

The development of force to change the velocity of the swimmer is of paramount concern during a race. In the present study, results from the land did not translate to the water. Specifically, the countermovement condition (dryland), while not yielding the largest force, did yield the largest impulse and resulted in the largest velocity at 2.5 m (Figure 2). This suggests that, on land, the countermovement is superior in the development of velocity, which was consistent with the in-water findings of a previous study [14]. The researchers believed that the results would have better translated to the water condition, had the swimmers been able to perform the two techniques with equal capability and the approach speed could have been controlled. However, the force profiles between the two push off techniques were not substantially different in water, as can be seen in Figure 3.

During the water trials, the swimmers were allowed to approach the wall in their usual manner but were then requested to perform the push off in the manner contrived on land (countermovement and no countermovement). In order to allow the wall to elicit a countermovement phase, (or eccentric portion of wall contact), the swimmer must approach the wall with enough velocity to have the wall force the swimmer to flex their knees [5,7,8]. However, it is hypothesized that during the countermovement push off, the swimmers not only used the wall to cause the knee flexion but also used drag to slow the body down before relying on the concentric movement to push off the wall. Relying on the drag to slow the body prevents the swimmer from storing as much elastic strain energy as on land, and thus, they cannot utilize the full benefit of the stretch shortening cycle.

The data collected from the water condition were not as clear as that of the land condition. In the land condition, the swimmers were pulled into the wall by elastic tubing that was stretched to the same amount for all trials. However, in the water condition, it was concluded that pulling the swimmers into the wall while on their backs was too far out of the comfort zone for the swimmers. Further, this study attempted to determine if the findings from the land condition translated not only to the water but to the flip turn motion as a whole. Specifically, we evaluated whether the participants could achieve the body positions that were requested and whether these body positions could produce the differences that were noted in the land condition. As indicated above, the results were not replicated. This absence of a transfer to the water condition could be the result of a lack of familiarity with the countermovement technique. Although the participants were allowed to practice the motion until they felt comfortable, distance from the wall prior to flip turn initiation may have hindered the eccentric loading component. To be considered a successful trial, the participants had to reach a knee flexion angle of 90°, but no regard was given to how much the wall contributed to that knee flexion. Therefore, the lack of significant differences between the peak force, impulse, and velocity of the countermovement turn versus the no countermovement turn suggests that the swimmers did not perform appreciatively different flip turn techniques when in the water.

The impulses determined in the present study are comparable to the previous literature (Table 1). It should be noted that direct comparisons to previous literature are difficult as most studies were done only in the water and included pull out phases of the swimming lap. For example, in 1999, Lyttle and colleagues noted that the participant who “sinks into the wall” had large push-off forces and high impulse [11]. This finding is partially supported by the results of this study. First, Lyttle et al.’s 1999 study was done completely in the water, and in the present study, no significant differences were noted between the peak force nor impulse across the two flip turn techniques in the water [11]. Furthermore, complicating this comparison is the land finding that larger forces were achieved during the extension flip-turn technique, but larger impulses were developed during the countermovement technique. It is also difficult to compare the velocities in the present study with previous literature as previous literature included swimming strokes and this project isolated only the push-off component of a lap of swimming. However, the velocities of the present study do fall within those reported by Blanksby et al. (1996b) [15].

It should be noted that there are several limitations to the current study. Firstly, the population included recreational and competitive swimmers. Though this was a within-subjects study, the differences in skill level should be noted. Secondly, a better method needs to be developed to investigate the differences in these two turn techniques in the water. Specifically, a device that could bring the swimming into the wall at controllable speeds would allow from a more comprehensive investigation of the relationship between the force developed and the drag encountered.

## 5. Conclusions

Superficially, it is tempting to suggest that the countermovement technique is superior to the no countermovement technique based solely on the land-based outcomes of this project. However, further research is required to determine whether swimmers can achieve an approach that allows for the beneficial performance of a countermovement or if the extra time spent in performing the countermovement technique is accounted for by the increased velocity in the water. A more gradual development of force associated with the countermovement technique may be beneficial as this would allow peak velocity to be achieved when the legs are more extended. Future research should focus on comparing these two flip turn techniques with swimmers that are proficient in one or the other. Overall, it is the assertion of the authors that the countermovement flip turn technique will prove to be superior. This is because it utilizes the stretch shortening cycle, allows for a more gradual development of velocity, thereby, encountering less drag, and ultimately further decreases the influence of drag by achieving maximum velocity when the swimmer is closest to streamline position.

## Figures and Tables

**Table 1:** Mean ± SD of the variables of interest in the present study and in water data from previous swimming turn push-off research (push-off technique unspecified). The data from the current study and previous work are presented here for comparison.

	Land			Water		
	Countermovement	No countermovement		Countermovement		No countermovement
	Present Study Data					
**Peak Perpendicular Force (N)**	854.5 ± 310.5*	1075.48 ± 274.9*		875.42 ± 342.7		972.68 ± 260.6
**Perpendicular Impulse (Ns)**	227.39 ± 56.4	198.8 ± 29.3		211.79 ± 65.2		235.14 ± 65.9
	Previous Research Data					
**Peak Perpendicular Force (N)**	535.9 ± 241.6 [3]		1189.6 ± 246.0 [11]		229.0 ± 70 (55% body weight) [13]	
**Perpendicular Impulse (Ns)**	150.6 ± 71.7 [3]		204.0 ± 54.9 [11]		55.6 ± 12.4 [13]	

* Significant difference between conditions (*p* = 0.018).
